# Three-Dimensional Localization Algorithm Based on Improved A* and DV-Hop Algorithms in Wireless Sensor Network

**DOI:** 10.3390/s21020448

**Published:** 2021-01-10

**Authors:** Xiaohu Huang, Dezhi Han, Mingming Cui, Guanghan Lin, Xinming Yin

**Affiliations:** 1 Department of Information Engineering, Shanghai Maritime University, Shanghai 201306, China; 201930310270@stu.shmtu.edu.cn (X.H.); cuimingming@stu.shmtu.edu.cn (M.C.); 201930310096@stu.shmtu.edu.cn (G.L.); 2 Department of Computer Science and Engineering, East China University of Science and Technology, 130 Meilong Road, Shanghai 200237, China; yinxm6@163.com; 3 The Third Research Institute of Ministry of Public Security, 76 Yueyang Road, Shanghai 200031, China

**Keywords:** wireless sensor networks (WSNs), Internet of Things (IoT), 3DDV-Hop, A* algorithm, NSGA-II, hop-count value, average distance per hop

## Abstract

In the traditional wireless sensor networks (WSNs) localization algorithm based on the Internet of Things (IoT), the distance vector hop (DV-Hop) localization algorithm has the disadvantages of large deviation and low accuracy in three-dimensional (3D) space. Based on the 3DDV-Hop algorithm and combined with the idea of A* algorithm, this paper proposes a wireless sensor network node location algorithm (MA*-3DDV-Hop) that integrates the improved A* algorithm and the 3DDV-Hop algorithm. In MA*-3DDV-Hop, firstly, the hop-count value of nodes is optimized and the error of average distance per hop is corrected. Then, the multi-objective optimization non dominated sorting genetic algorithm (NSGA-II) is adopted to optimize the coordinates locally. After selection, crossover, mutation, the Pareto optimal solution is obtained, which overcomes the problems of premature convergence and poor convergence of existing algorithms. Moreover, it reduces the error of coordinate calculation and raises the localization accuracy of wireless sensor network nodes. For three different multi-peak random scenes, simulation results show that MA*-3DDV-Hop algorithm has better robustness and higher localization accuracy than the 3DDV-Hop, PSO-3DDV-Hop, GA-3DDV-Hop, and N2-3DDV-Hop.

## 1. Introduction

Nowadays, wireless sensor network (WSN) is a research hotspot in the field of Internet of Things (IoT), which is composed of several micro sensor nodes with data collection, analysis and processing capabilities as well as information sending and receiving capabilities [1]. Owing to the deployment location of sensor nodes in WSN can be changed at any time, it can monitor some specific areas flexibly [2,3].

In practical applications, most wireless sensor nodes are deployed in 3D space scene, such as forest fire warning [4]. The node monitors the fire source and transmits the data to the user, then the user can quickly take the fastest rescue speed to implement the fire fighting action and minimize the loss [5]. In the battlefield behind the enemy, real-time monitoring of the enemy’s weapons and high-tech equipment area, the direction of moving vehicles and the deployment of troops can accurately grasp the enemy’s trend [6]. For the application of natural water pipeline leakage, it is necessary to know the leakage location accurately to make targeted remedial measures [7]. In some special cases, when it is inconvenient, or the staff are unable to enter the monitoring area to carry out survey work, the unique advantages and characteristics of WSN can be used to quickly monitor the surrounding environmental heat, red ultraviolet, sonar, vital signs and seismic wave signals [8]. The detected physical information, including temperature, light, air humidity, pressure, soil composition, speed, size and movement direction of moving objects, is given to users for observation and processing. The primary function of WSN is to quickly and accurately locate the source of events, which is also the most basic problem in practical application [9].

WSN node localization algorithm can be divided into direct-ranging-method and indirect- ranging-method [10]. The direct-ranging-method utilizes additional hardware to measure the angle, distance and signal strength between nodes, and then calculates the localization information of the nodes by using the obtained data [11]. Typical direct-ranging-methods include strength indication of received signal (RSSI) [12,13], time difference of arrival (TDOA) [14], time of arrival (TOA) [15,16], angle of arrival (AOA) [17,18], and so forth. Indirect-ranging-method mainly uses geometric relationship between nodes, hop-count value and energy loss between nodes, network connectivity and other aspects to estimate the location of unknown nodes [19]. It includes DV-Hop [20], perfect point in triangulation test (APIT) [21,22], centroid algorithm [23,24], and so forth.

The DV-Hop algorithm is not complicated. In different networks, its localization results are relatively accurate and reasonable, and the network robustness is also strong  [25]. However, there is a large localization error in the random sensor networks with uneven density [26]. The larger the minimum hop-count value from the localization node to the beacon node, the greater the cumulative error value [27]. On account of the error is directly proportional to the hop-count value, when the hop-count value is large, the localization result will be inaccurate if the estimated distance with large error is used to calculate the node localization coordinates [28,29].

Aiming at the shortcomings of existing DV-Hop algorithms, this paper proposes a localization algorithm(MA*-3DDV-Hop) based on 3D space. MA*-3DDV-Hop algorithm can not only adapt to 2D space node localization, but also can be applied to 3D space node accurate localization. In MA*-3DDV-Hop algorithm, the hop-count value of nodes is optimized to improve the error of calculating the average distance per hop. Then, the multi-objective NSGA-II is adopted to optimize the coordinates locally. After selection, crossover, mutation, the Pareto optimal solution is obtained, which overcomes the problems of premature convergence and poor convergence of existing algorithms. The above steps reduce the error of coordinate calculation and promotes the localization accuracy of wireless sensor network nodes.

The main contributions of this paper are as follows:

- The improved A* algorithm is used to optimize the hop-count value of the localization node in the network. The original hop-count value and deviation angle are normalized to realize the intelligent selection of the best node to be hopped, which makes the hop-count value path between each node tend to be straight line, and the new hop-count value is obtained through iterative calculation.
- The average distance per hop is corrected. The normalized values of the original hop-count value and the deviation angle are used as the correction factors to correct the estimated distance per hop, so as to further diminish the error of the average distance per hop.
- The NSGA-II algorithm is used to refine the estimated coordinates iteratively in the local area to further enhance the accuracy. The coordinate value obtained by the least square method formula is used as reference, and the best search target range is set. Through selection, crossover, mutation operation, to find the best chromosome, get the Pareto optimal solution, and finally solve the optimal coordinate value.
- The MA*-3DDV-Hop algorithm is compared with the existing algorithms in three different 3D multi-peak simulation environments. The experimental results show that compared with 3DDV-Hop, PSO-3DDV-Hop [30], GA-3DDV-Hop [31] and N2-3DDV-Hop [31], MA*-3DDV-Hop algorithm has smaller localization error and stronger stability, and is more suitable for application scenes with higher localization accuracy and stability requirements.

The rest of this paper is structured as follows: In Section 2, we introduce the related work of the proposed algorithm. In Section 3, the classical DV-Hop and A* algorithms are described. In Section 4, the MA*-3DDV-Hop algorithm proposed in this paper is explained. In Section 5 and Section 6, the experimental results and performance evaluation are analyzed. Finally, the conclusion of this paper and the future work direction are presented in Section 7.

## 2. Related Work

DV-Hop algorithm is simple, widely used, stable and easy to expand [26]. Many researchers put forward different ideas to enhance the localization accuracy of the algorithm: Tang et al. used TDOA location feature to quantify the hop value of nodes in DV-Hop location algorithm to achieve a stable effect [32]. Li et al. suggested increasing the communication radius to update the minimum hop-count value of unknown nodes to a smaller hop-count value, which solves the problem that the actual distance difference of the same hop-count value to a certain extent [33]. Cai et al. proposed a new algorithm (N2-3DDV-Hop) to modify the average distance per hop and to optimize the error combined with NSGA-II algorithm [31]. Han et al. applied a differential evolution localization algorithm based on DV-Hop to modify the average distance per hop [34]. Liu et al. used a hybrid localization algorithm of APIT and DV-Hop, which enhances the localization accuracy and node coverage [35]. Singh et al. improved DV-Hop method and applied meta heuristic technology to overcome the localization error [36]. Chai et al. proposed a new parallel whale optimization algorithm, which can get better results than some existing intelligent computing algorithms [37]. Cheng et al. proposed to use the coincidence particle swarm optimization method to modify the average distance per hop [38]. In addition, Xiao et al. adopted to use particle swarm optimization algorithm for global optimization after the least square method is used to calculate the coordinates [39].

All the above improved algorithms only carry out unilateral error correction or coordinate optimization, but ignore the gradual error correction, which results in the accumulation of errors layer by layer and unsatisfactory localization accuracy. This is because DV-Hop algorithm locates unknown nodes based on distance vector information and network connectivity, instead of directly measuring the distance between nodes [40]. The error of the algorithm itself is mainly caused by the following aspects:

- In traditional DV-Hop algorithm or improved DV-Hop algorithm, nodes are generally distributed randomly. If the propagation path and straight line between the two nodes have large difference and lack of consistency, then the hop-count value calculated in the first stage will produce large error.
- In the second stage, there is a big error when the original hop-count value obtained in the first stage is substituted into the calculated average distance per hop. Then the average distance per hop with error is used to estimate the distance between the unknown node and the beacon node, which will inevitably affect the localization accuracy.
- Due to the accumulation of errors in the first stage and the second stage, the deviation between the calculated coordinates in the third stage and the real coordinates is large. The localization results are not ideal.

To sum up the above, this paper first combines the idea of A* algorithm to gradually correct the error of hop-count value in the first step and the average distance per hop in the second step, so as to minimise the Cumulative error and strengthen the localization accuracy to a certain extent. And then, in the third stage, the estimated coordinate value calculated by the least square method is taken as the target to establish the optimal search target range and multi-objective function. The optimal solution is obtained by local optimization combined with NSGA-II algorithm, that is, the search range is narrowed from the global search to the local range of the estimated coordinates to find the optimal coordinate value, which greatly decreases the coordinate error.

## 3. Three-Dimensional Distance Vector-Hop (3DDV-Hop) and A* Algorithm

This section will introduce 3DDV-Hop algorithm and A* algorithm in detail.

### 3.1. 3DDV-Hop Algorithm

3DDV-Hop coordinates calculation is divided into the following three steps:

Stept 1: Through Flooding, the Hop-count Value is Estimated

In this step, beacon nodes in wireless sensor networks broadcast packets containing their own number, location information and hop-count value by flooding, and the hop-count value is set to zero [41,42]; after receiving the data packet of the beacon node, the neighbor node of the beacon node adds one hop-count value in the packet and forwards it to the neighbor node of the next hop of the neighbor node [43,44]; the node receiving the packet selects the packet with the minimum hop-count value from a number of packets with the same number and saves it, adds the hop-count value in the selected packet by one, and forwards the packet to the next hop neighbor node of the node [45,46]. This is repeated until the end of flooding. The hop-count value of the packet saved by any node in the network is the minimum hop-count value between the node and the corresponding beacon node [47].

Stept 2: Distance Estimation Calculation

In this step, the average distance per hop between beacon nodes is calculated by using Equation (1) in 3D cyberspace.

(1) $$H\mathrm{op}size_{i}\_pre=\frac{\sum_{i\neq j}\sqrt{{\left(x_{i}-x_{j}\right)}^{2}+{\left(y_{i}-y_{j}\right)}^{2}+(z_{i}-z_{j}{)}^{2}}}{\sum_{i\neq j}Hop_{i}^{j}\_pre},$$

where $Hop_{i}^{j}\_pre$ is the minimum hop-count value from beacon node *i* to beacon node *j* in the network, i≠j and i,j∈[1,N], $\left(x_{i},y_{i},z_{i}\right)$ and $\left(x_{j},y_{j},z_{j}\right)$ represent the coordinates of beacon nodes *i* and *j* respectively, while $H\mathrm{op}size_{i}\_pre$ represents the average distance per hop from beacon node *i* to all other beacon nodes *j* in the network.

Then, the estimated distance between unknown node *p* and beacon node *j* is calculated. The formula is as follows:

(2) $$d_{pi}=Hopsize_{i}\_pre\times Hop_{p}^{i}\_pre,$$

where p∈[1,W], *W* is the number of unknown nodes, i∈[1,N], and *N* is the number of beacon nodes.

Stept 3: Calculating Coordinates

In this step, the estimated distance $d_{pi}$ between the unknown node and each beacon node is calculated according to Equation (2). Let the coordinates of unknown node *p* and beacon node *i* be $\left(X_{p},Y_{p},Z_{p}\right)$ and $\left(x_{i},y_{i},z_{i}\right)$ respectively, i∈[1,N]. According to the estimated distance between unknown node *p* and beacon node *i*, the equations are listed [48]:

(3) $$\left\{\begin{array}{c} \begin{array}{c} {\left(X_{1}-X_{p}\right)}^{2}+{\left(Y_{1}-Y_{p}\right)}^{2}+{\left(Z_{1}-Z_{p}\right)}^{2}=d_{p1}^{2}\\ {\left(X_{2}-X_{p}\right)}^{2}+{\left(Y_{2}-Y_{p}\right)}^{2}+{\left(Z_{2}-Z_{p}\right)}^{2}=d_{p2}^{2} \end{array}\\ \begin{array}{c} ..............\\ {\left(X_{N}-X_{p}\right)}^{2}+{\left(Y_{N}-Y_{p}\right)}^{2}+{\left(Z_{N}-Z_{p}\right)}^{2}=d_{pN}^{2} \end{array} \end{array}\right.$$

The equations are solved:

(4) $$\left[\begin{array}{c} X_{p}\\ Y_{p}\\ Z_{p} \end{array}\right]={\left(A^{T}A\right)}^{-1}A^{T}b,$$

where T stands for transposition,

(5) $$A=\left[\begin{array}{ccc} 2(X_{1}-X_{N}) & 2(Y_{1}-Y_{N}) & 2(Z_{1}-Z_{N})\\ 2(X_{2}-X_{N}) & 2(Y_{2}-Y_{N}) & 2(Z_{2}-Z_{N})\\ .... & .... & ....\\ 2(X_{N-1}-X_{N}) & 2(Y_{N-1}-Y_{N}) & 2(Z_{N-1}-Z_{N}) \end{array}\right]$$

(6) $$b=\left[\begin{array}{c} X_{1}^{2}-X_{N}^{2}+Y_{1}^{2}-Y_{N}^{2}+Z_{1}^{2}-Z_{N}^{2}+d_{pN}^{2}-d_{p1}^{2}\\ X_{2}^{2}-X_{N}^{2}+Y_{2}^{2}-Y_{N}^{2}+Z_{2}^{2}-Z_{N}^{2}+d_{pN}^{2}-d_{p2}^{2}\\ ......\\ X_{N-1}^{2}-X_{N}^{2}+Y_{N-1}^{2}-Y_{N}^{2}+Z_{N-1}^{2}-Z_{N}^{2}+d_{pN}^{2}-d_{pN-1}^{2}. \end{array}\right]$$

### 3.2. A* Algorithm

A* algorithm is not only the most effective direct search method for solving the shortest path, but also a common heuristic algorithm for many other problems. Therefore, a good heuristic function based on environment selection is crucial [49].

**Figure 1:**
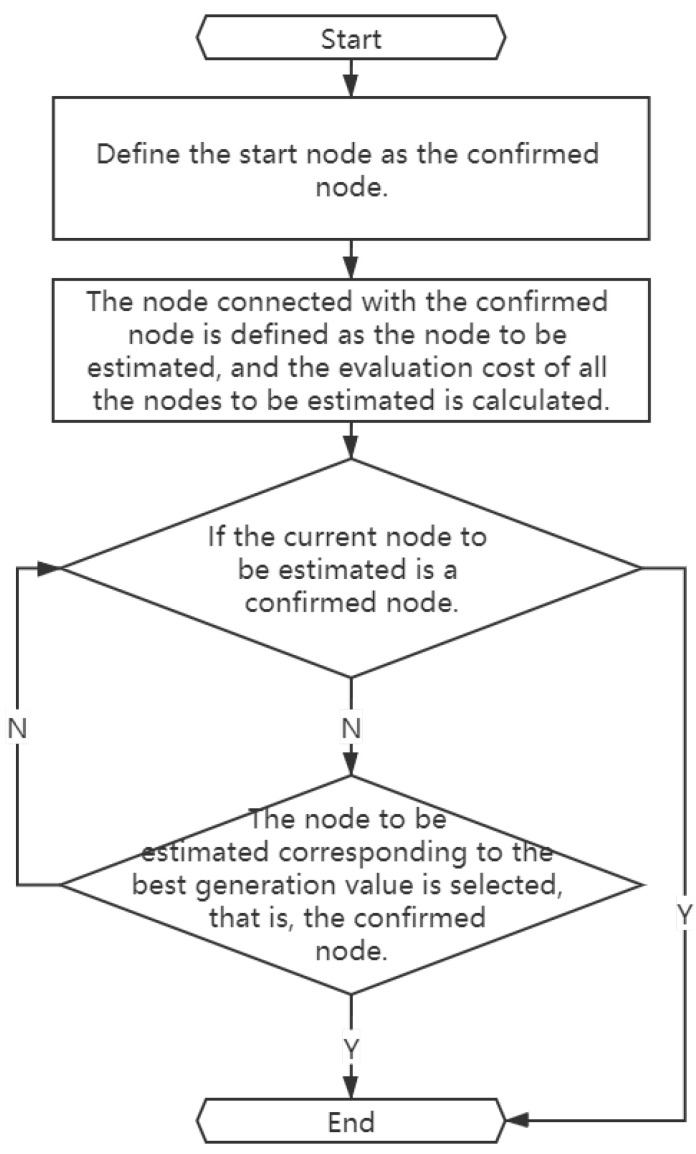
Logic diagram of A* algorithm.

The formula of A* algorithm is shown as follows:

(7) f(n)=g(n)+h(n)

where f(n) is the cost estimation from the initial state to the target state via state *n*, g(n) is the actual cost from the initial state to the state *n* in the state space, and h(n) is the estimated cost of the best path from state *n* to target state [50]. The overall flow chart of A* algorithm is shown in Figure 1, and the related steps are as follows [50]:

1. Define the start node as an existing node.
2. The node connected with the confirmed node is defined as the node to be estimated, and the evaluation cost of all the nodes to be estimated is calculated.
3. Determine: If the current node to be estimated is a confirmed node, it will stop immediately; otherwise, the execution will continue.
4. The node to be estimated corresponding to the best generation value is selected as the confirmed node.
5. Otherwise, go back to Step 3.

## 4. MA*-3DDV-Hop Algorithm

In this section, the DV-Hop model described in Section 3 is improved combined with A* algorithm to gradually decrease the error, and NSGA-II algorithm is used to optimize the final cumulative error.

### 4.1. Improved 3DDV-Hop Using A* Algorithm

As mentioned above, DV-Hop algorithm locates unknown nodes through distance vector information and network connectivity, and does not directly measure the distance between nodes, resulting in errors. In this paper, A* algorithm is used to optimize the hop-count path to cut down the error, and the average distance per hop is corrected by normalizing the original hop-count value and deviation angle degree as the error coefficient. The specific steps of MA*-3DDV-Hop algorithm are as follows:

Stept 1: Optimization of the Hop-count Value

**Figure 2:**
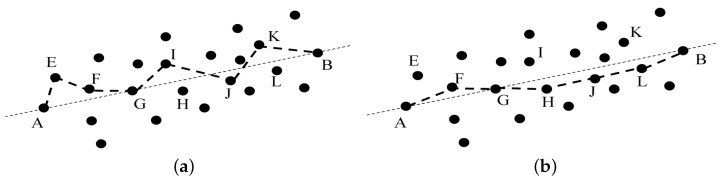
(**a**) Node path before optimization. (**b**) Optimized node path.

Firstly, the original hop-count value $Hop_{i}^{j}\_pre$ is obtained by using the first flooding path and Equation (1), as shown in Figure 2a.

Secondly, combined with the idea of A* algorithm, the cost evaluation function from unknown node *x* to beacon node is established:

(8) $$F_{j}\left(x\right)=g_{k}\left(x\right)+h_{j}\left(k\right),$$

where $F_{j}\left(x\right)$ is the total cost from unknown node *x* to beacon node *j*, node *k* is any node between beacon node *x* and beacon node *j*, $g_{k}\left(x\right)$ is the cost from unknown node *x* to unknown node *k*, $h_{j}\left(k\right)$ is the evaluation from unknown node *k* to beacon node *j*, j∈[1,N],x,k∈[1,W].

**Figure 3:**
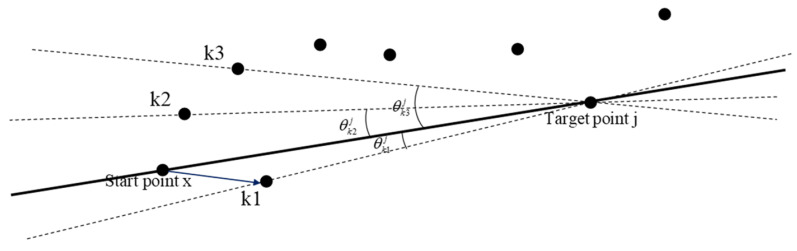
Diagram of deviation angle of unknown node *k*.

The original hop-count value $Hop_{i}^{j}\_pre$ and the unknown node deviation angle degree $\theta_{{}_{k}}^{j}$ (the angle between Euclidean distance from node *x* to node j and Euclidean distance from unknown node *k* to node *j* can be obtained by AOA and other related technologies, as shown in Figure 3) are normalized. The adjustable factors $\varpi_{1}$, $\varpi_{2}$, and the angle factor θ are set. The discriminant function is as follows:

(9) $$h_{j}\left(k\right)=\varpi_{1}\times \frac{Hop_{k}^{j}\_pre-\min\left(Hop_{i}^{j}\_pre\right)}{\max\left(Hop_{i}^{j}\_pre\right)-\min(Hop_{i}^{j}\_pre)}+\varpi_{2}\times \frac{\theta_{{}_{k}}^{j}}{\theta }.$$

Finally, a new path is obtained by flooding, and the optimized hop-count value is $Ho{p_{i}}^{j}\_A*$, as shown in Figure 2b.

Stept 2: Correction of Average Distance Per Hop and Calculation of the Estimated Distance

First of all, the average distance per hop $H\mathrm{op}size_{i}\_A*$ between beacon nodes is calculated:

(10) $$H\mathrm{op}size_{i}\_A*=\frac{\sum_{i\neq j}\sqrt{{\left(x_{i}-x_{j}\right)}^{2}+{\left(y_{i}-y_{j}\right)}^{2}+(z_{i}-z_{j}{)}^{2}}}{\sum_{i\neq j}Ho{p_{i}}^{j}\_A*},$$

where $\left(x_{i},y_{i},z_{i}\right)$, $\left(x_{j},y_{j},z_{j}\right)$ represents the coordinates of beacon nodes *i* and *j* respectively, $Ho{p_{i}}^{j}\_A*$ represents the hop-count value between beacon nodes *i* and *j*, i,j∈[1,N].

Secondly, using Equation (11) to correct the error, the corrected average distance per hop is obtained:

(11) $$H\mathrm{op}size_{i}\_A*\_M=H\mathrm{op}size_{i}\_A*+\lambda f^{j}\left(i\right),$$

where $f^{j}\left(k\right)$ is the cost function from unknown node *k* to beacon node *j*, and λ is the correction factor.

On the basis of solving the modified average distance per hop Hopsize_A*_M_Ave, the average distance per hop based on the whole network is solved by Equation (12).

(12) $$H\mathrm{op}size\_A*\_M\_A\mathrm{ve}=\frac{\sum_{i=1}^{N}H\mathrm{op}size\_A*\_M}{N}.$$

Finally, the estimated distance $d_{uj}$ between beacon node *j* and unknown node *u* is calculated by using Equation (13):

(13) $$d_{uj}=H\mathrm{op}size\_A*\_M\_A\mathrm{ve}\times Hop_{uj}\_A*,$$

where u∈[1,W],j∈[1,N].

### 4.2. NSGA-II Algorithm

**Figure 4:**
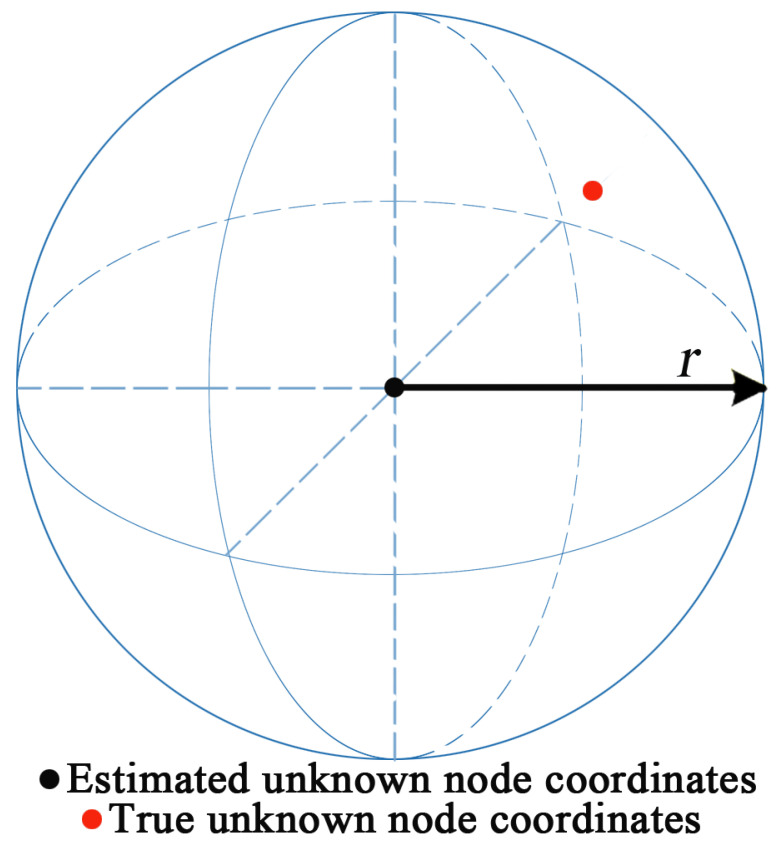
Schematic diagram of local optimization of NSGA-II algorithm.

In this stage, the multi-objective NSGA-II algorithm is used to locally optimize the initial estimated unknown node coordinates, as shown in Figure 4. On account of the error *∂* of estimated distance and true distance between unknown node and beacon node, the problem of solving unknown node coordinate can be transformed into solving the minimum value problem of Equation (14).

(14) $$f\left(x,y,z\right)=\min\left(\sum_{j=1}^{N}\left|\sqrt{{\left(x_{u}-x_{j}\right)}^{2}+{\left(y_{u}-y_{j}\right)}^{2}+(z_{u}-z_{j}{)}^{2}}-d_{j}\right|\right),$$

where $\left(x_{u},y_{u},z_{u}\right)$ is the coordinate of unknown node, $\left(x_{j},y_{j},z_{j}\right)$ is the coordinate of beacon node, and $d_{j}$ is the estimated distance from unknown node *u* to beacon node *j*.

On the basis of the estimated unknown node coordinates $\left(X_{u},Y_{u},Z_{u}\right)$, the multi-objective optimization function of NSGA-II is established as follows:

(15) $$\min f_{1}\left(x_{u},y_{u},z_{u}\right)=\left|\sqrt{{\left(x_{u}-x_{j1}\right)}^{2}+{\left(y_{u}-y_{j1}\right)}^{2}+(z_{u}-z_{j1}{)}^{2}}-d_{uj1}\right|$$

(16) $$\min f_{2}\left(x_{u},y_{u},z_{u}\right)=\left|\sqrt{{\left(x_{u}-x_{j2}\right)}^{2}+{\left(y_{u}-y_{j2}\right)}^{2}+(z_{u}-z_{j2}{)}^{2}}-d_{uj2}\right|$$

(17) $$s.t.\left\{\begin{array}{c} X_{u}-r<x_{u}<X_{u}+r\\ Y_{u}-r<y_{u}<Y_{u}+r\\ Z_{u}-r<z_{u}<Z_{u}+r, \end{array}\right.$$

where $f_{1}\left(x_{u},y_{u},z_{u}\right)$ and $f_{2}\left(x_{u},y_{u},z_{u}\right)$ are respectively the first objective function and the second objective function in the multi-objective model; $d_{uj1}$ and $d_{uj2}$ are the distances between the two beacon nodes nearest to the unknown node *u*, j1,j2∈N; *r* is the constraint radius of local optimization.

### 4.3. The Proposed MA*-3DDV-Hop Algorithm

**Figure 5:**
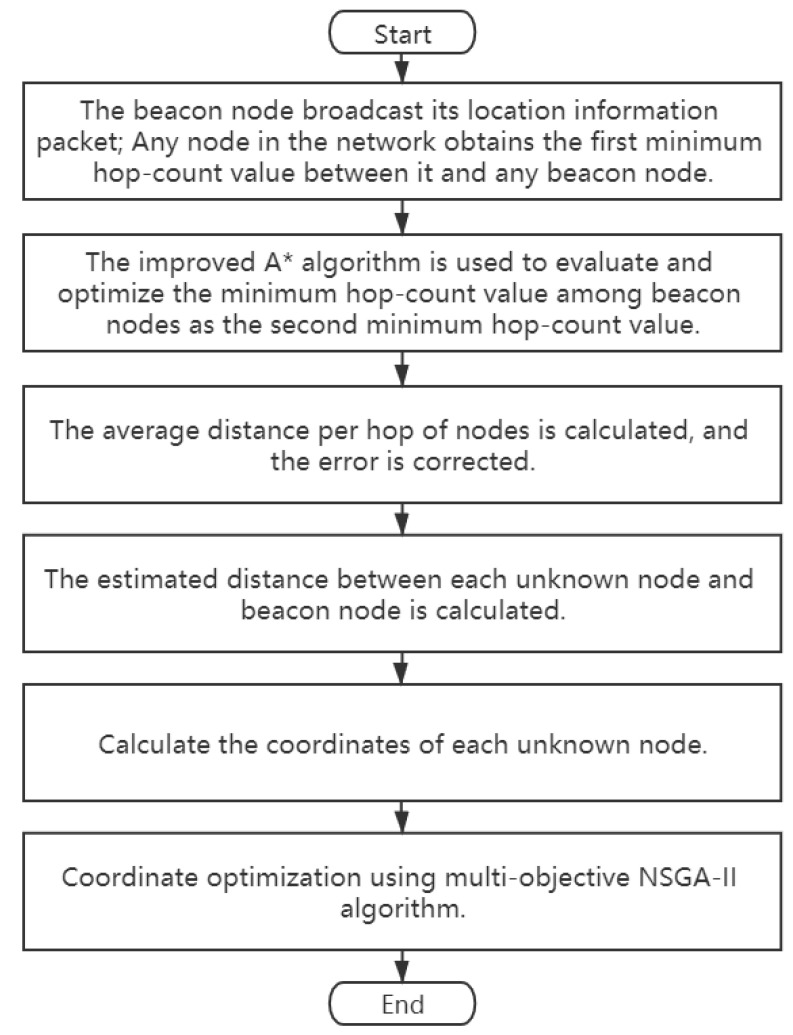
Logic diagram of MA*-3DDV-Hop algorithm.

This paper presents the MA*-3DDV-Hop algorithm based on DV-Hop model, which combines A* algorithm with NSGA-II algorithm. Figure 5 helps us better understand the complete implementation steps of MA*-3DDV-Hop algorithm. Algorithm 1 is the pseudo code of MA*-3DDV-Hop algorithm.
**Algorithm 1** The procedure of MA*-3DDV-Hop. **Initialization:** The number of nodes M, the beacon nodes N, communication radius Rc **Input:** Parameter settings of MA*-3DDV-Hop and the experimental area; Parameter settings of MA*-3DDV-Hop: see Table 1; Experimental area is 100 × 100 × 100 $m^{3}$ 1: Generate simulated 3D random topology scene; 2: Calculate the hop-count value $Hop_{i}^{j}\_pre$ according to the shortest path algorithm; 3: Calculate the hop-count value $Ho{p_{i}}^{j}\_A*$ according to the shortest path algorithm again combination with Equations (7) and (8); 4: **for** k = 1 to M **do** 5:  **for** i = 1 to M **do** 6:   **for** j = 1 to M **do** 7:    **if** h(x) < min(h(x)) **then** 8:     Min(h(x)) = h(x); 9:     Min(h(x))_index=points(i);; 10:    **end if** 11:   **end for**
 12:   F(x) = g(x) + min(h(x)); 13:   num = num + 1; 14:  **end for**
 15: **end for** 16: Calculate the average distance per hop; $H\mathrm{op}size_{i}\_A*\_M\_A\mathrm{ve}$, according to Equations (9)–(11); 17: Calculate the unknown distances; According to Equation (12); 18: Calculate the coordinate; 19: Calculate the fitness of objective function using Equation (14), fast non-dominated sort, selection, crossover, mutation. Execution NSGA-II algorithm see Table 1; 20: lb = S(i,:)-r; 21: ub = S(i,:)+r; 22: ………. 23: end

## 5. Experiment and Result Analysis

In this section, we will establish the simulation experiment for the algorithm proposed in Section 4. Firstly, the experimental environment is introduced. Then, the feasibility and evaluation criteria of the scheme are explained. Finally, the experimental results are analyzed.

### 5.1. Experimental Environment

In this experiment, the MA*-3DDV-Hop algorithm is simulated and tested by running MATLAB 2018b on a computer configured with Intel(R) Core(TM) I7-8700 CPU @3.20ghz processor, 8 GB RAM, and Windows 10 operating system. In order to obtain good experimental results, each experiment is run independently for 50 times. MA*-3DDV-Hop algorithm is compared and analyzed with 3DDV-Hop, PSO-3DDV-Hop, GA-3DDV-Hop, and N2-3DDV-Hop.

**Figure 6:**
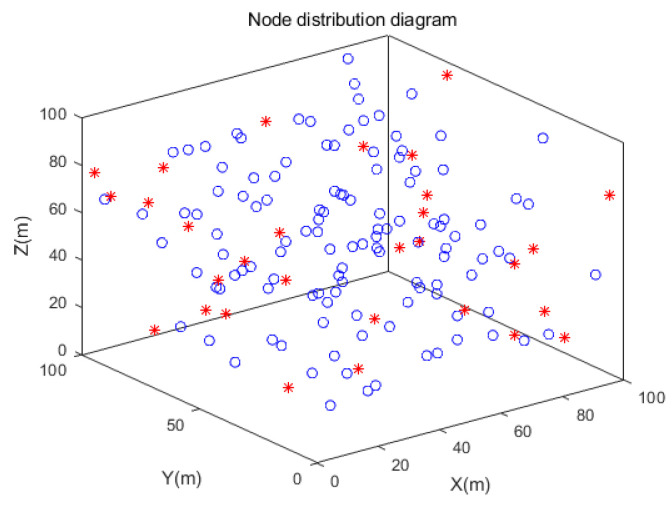
Simulation effect of random topology scene.

**Figure 7:**
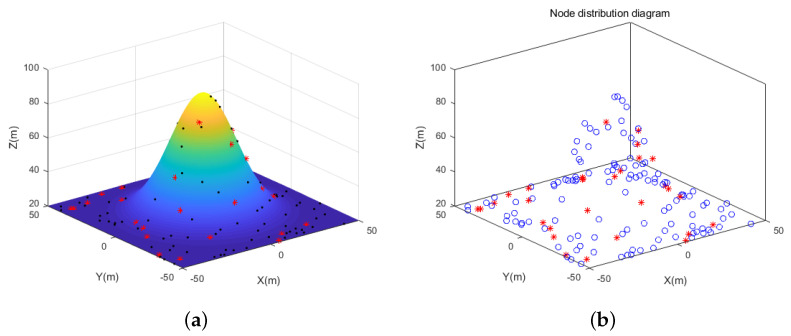
(**a**) Simulation effect of unimodal random topology scene. (**b**) Rendering of node distribution in unimodal random topology scene.

**Figure 8:**
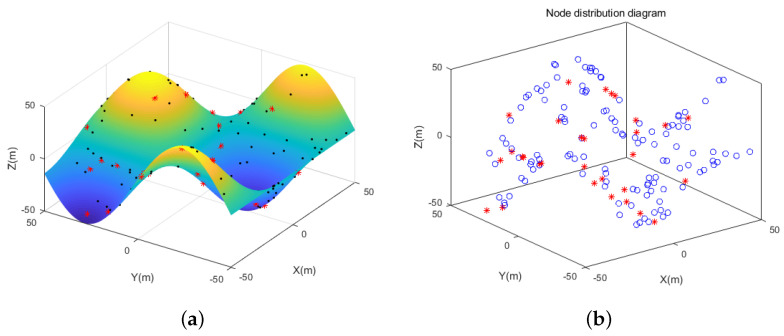
(**a**) Simulation effect of multi-peak random topology scene. (**b**) Rendering of node distribution in multi-peak random topology scene.

The simulation experiments are carried out in three different 3D scene conditions: random topology scene, unimodal random topology scene, and multi-peak random topology scene. The circle nodes represent unknown nodes, and the star nodes represent beacon nodes, as shown in Figure 6, Figure 7 and Figure 8. In the random topology scene, all nodes are randomly distributed in 100 m × 100 m × 100 m space region. In the unimodal random topology scene, all nodes are randomly distributed on the unimodal model based on the 100 m × 100 m × 100 m space region. While in the multi-peak random topology scene, all nodes are randomly distributed on the multi-peak model based on the 100 m × 100 m × 100 m space region. Other relevant parameters are shown in Table 1.

### 5.2. Feasibility of the Scheme

**Figure 9:**
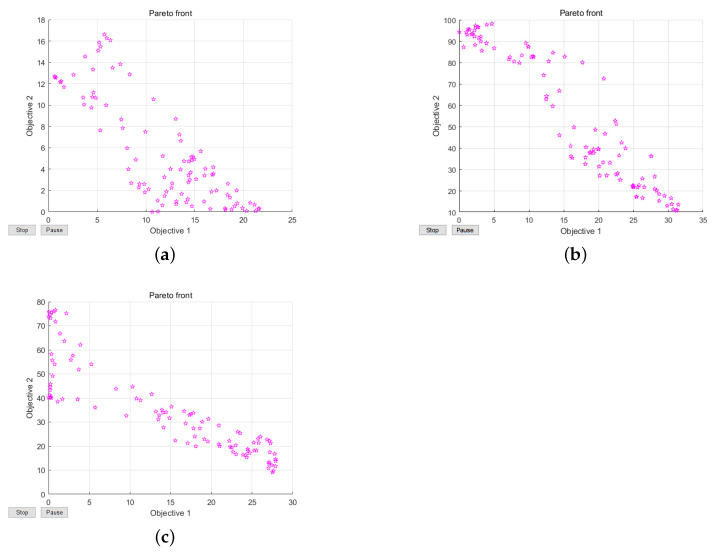
(**a**) Pareto frontier graph of random topological scene. (**b**) Pareto frontier graph of unimodal topological scene.(**c**) Pareto frontier graph of multi-modal topological scene.

Figure 9 shows the multi-objective Pareto frontier diagrams of random topology scene, unimodal random topology scene, and multi-modal random topology scene. It can be seen from Figure 9 that the inverse relationship between Pareto model functions in random topology scene, unimodal random topology scene, and multimodal random topology scene becomes more and more remarkable. With the increase of three kinds of spatial complexity, the optimization scale based on multi-objective Pareto model is also increasing. Owing to the growth of three kinds of spatial complexity, the optimization scale of multi-objective Pareto model is also raising, which better verifies the feasibility and correctness of multi-objective model function.

**Figure 10:**
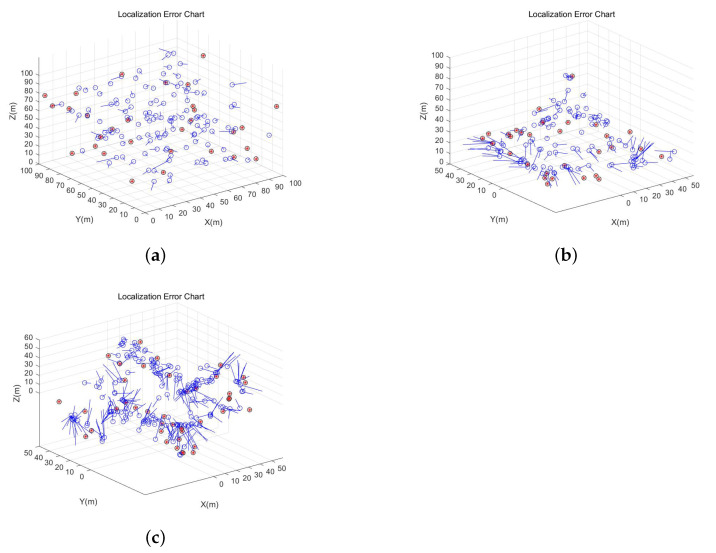
(**a**) The error graph of random topological scene. (**b**) The error graph of unimodal random topological scene.(**c**) The error graph of muti-peak random topological scene.

In order to verify the global feasibility of the scheme, the whole algorithm is simulated in three different scenes, and the error effect diagram is obtained, as shown in Figure 10. In the random topology scene, it clearly shows that all nodes are randomly deployed in the corresponding experimental area. In the unimodal and multi-peak topology scene, all nodes are randomly and evenly deployed on the complex terrain, which better simulates the real scene.

### 5.3. Evaluation Criteria for Tests

After the localization algorithm is completed, the evaluation error of unknown node *u* is as follows:

(18) $$Er_{u}=\sqrt{{\left(x_{{}_{u}}^{e}-x_{{}_{u}}^{t}\right)}^{2}+{\left(y_{{}_{u}}^{e}-y_{{}_{u}}^{t}\right)}^{2}+{{(z}_{{}_{u}}^{e}-z_{{}_{u}}^{t})}^{2}}.$$

Average localization error (*ALE*) as the evaluation standard of this paper:

(19) $$ALE=\sum_{u=1}^{W}\frac{\sqrt{{\left(x_{{}_{u}}^{e}-x_{{}_{u}}^{t}\right)}^{2}+{\left(y_{{}_{u}}^{e}-y_{{}_{u}}^{t}\right)}^{2}+{{(z}_{{}_{u}}^{e}-z_{{}_{u}}^{t})}^{2}}}{W\times Rc},$$

where $\left(x_{{}_{u}}^{e},y_{{}_{u}}^{e},z_{{}_{u}}^{e}\right)$ is the estimated coordinates finally calculated by the localization algorithm. $\left(x_{u}^{t},y_{{}_{u}}^{t},z_{{}_{u}}^{t}\right)$ is the true coordinate. ALE is the average localization error, *W* is the number of unknown nodes, and Rc is the communication radius.

### 5.4. Analysis of Experimental Results

In this section, we study the influence of communication radius, the number of nodes, and the number of beacon nodes on ALE in three different topological scenes.

#### 5.4.1. The Influence of Communication Radius

**Figure 11:**
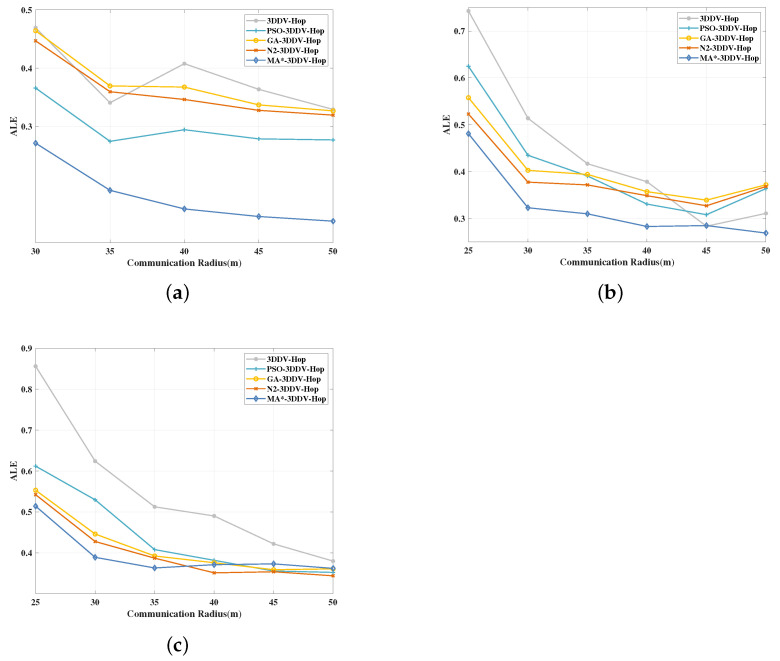
(**a**) The effect of communication radius on ALE in a random topology scene. (**b**) The effect of communication radius on ALE in unimodal random topology scene.(**c**) The effect of communication radius on ALE in a multi-peak random topology scene.

**Figure 12:**
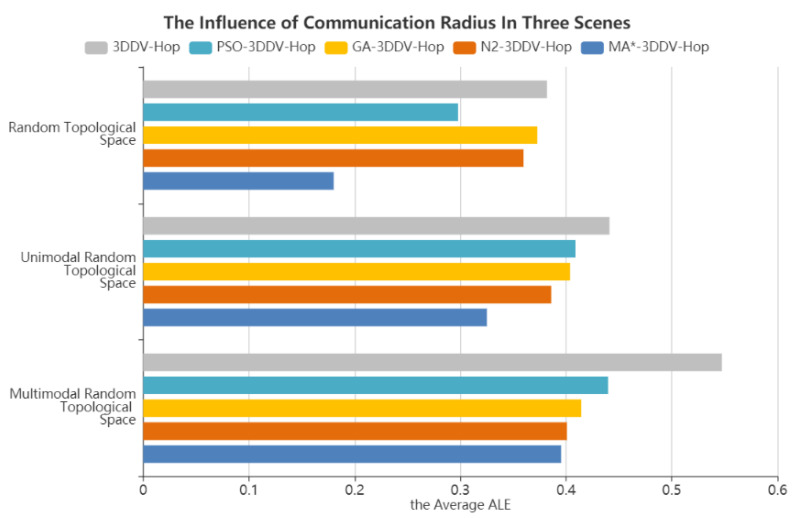
The average average localization error (ALE) of five algorithms in three scenes.

In the three scenes, the communication radius of the nodes is set to a distance between 25 m and 50 m, the number of deployed nodes is 130, and the number of beacon nodes is 25. The experimental results are shown in Figure 11 and Figure 12.

It can be seen from Figure 11 that, in the three network topology scenes, the ALE of the five localization algorithms mentioned above decreases gradually with the increase of the communication radius in the network. Among the five algorithms, MA*-3DDV-Hop has notable advantages in the scenes of random topology and unimodal random topology, and ALE value always keeps the minimum. In the multi-peak random topology scene, we can see that due to the complex terrain, MA*-3DDV-Hop is less affected by the increase of communication radius, and ALE value changes slowly.

Figure 12 shows the influence of the node communication radius on the average ALE of the five algorithms based in the three scenes. As can be seen from the figure, compared with 3DDV-Hop, the average ALE of PSO-3DDV-Hop, GA-3DDV-Hop, N2-3DDV-Hop, and MA*-3DDV-Hop in random topology scene are reduced by 22%, 2.3%, 5.8% and 52% respectively. Compared with 3DDV-Hop, the average ALE of PSO-3DDV-Hop, GA-3DDV-Hop, N2-3DDV-Hop, and MA*-3DDV-Hop reduces by 7.3%, 8.4%, 12% and 26% respectively in the unimodal random topology scene. In the multi-peak random topology scene, the average ALE of PSO-3DDV-Hop, GA-3DDV-Hop, N2-3DDV-Hop, and MA*-3DDV-Hop reduces by 19%, 24%, 26%, and 27%, respectively, compared with 3DDV-Hop.

As shown in Figure 11 and Figure 12, the localization error of wireless sensor network nodes can be cutted down by increasing the communication radius of nodes in three different scenes. In the scenes of random topology and unimodal random topology, as the communication radius increases, MA*-3DDV-Hop has significant advantages over other algorithms in terms of ALE and average ALE; in multi-peak topology scene, ALE of MA*-3DDV-Hop is less affected by the increase of communication radius, but the average ALE is better than other algorithms. The results show that the MA*-3DDV-HOP algorithm has better robustness and localization performance when the communication radius is increased.

#### 5.4.2. The Influence of the Number of Nodes

**Figure 13:**
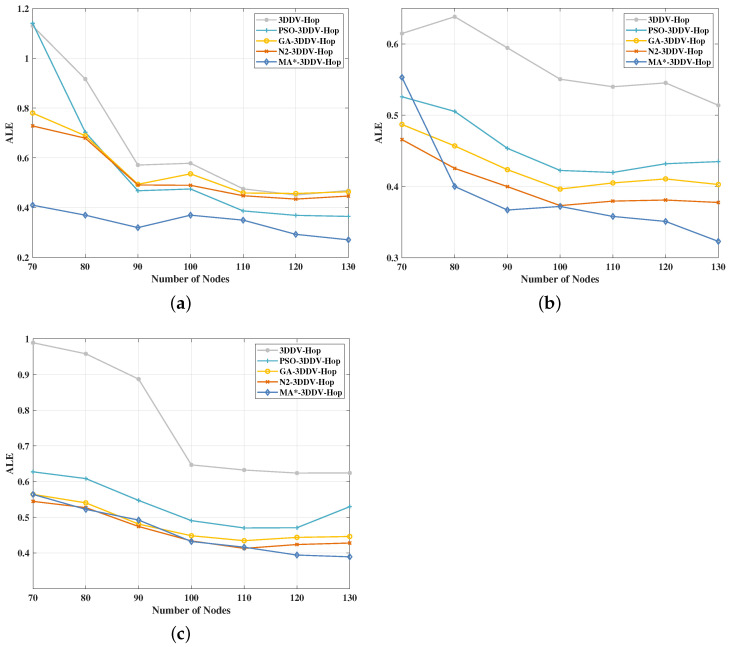
(**a**) The effect of the number of nodes on ALE in a random topology scene. (**b**) The effect of the number of nodes on ALE in unimodal random topology scene.(**c**) The effect of the number of nodes on ALE in a multi-peak random topology scene.

**Figure 14:**
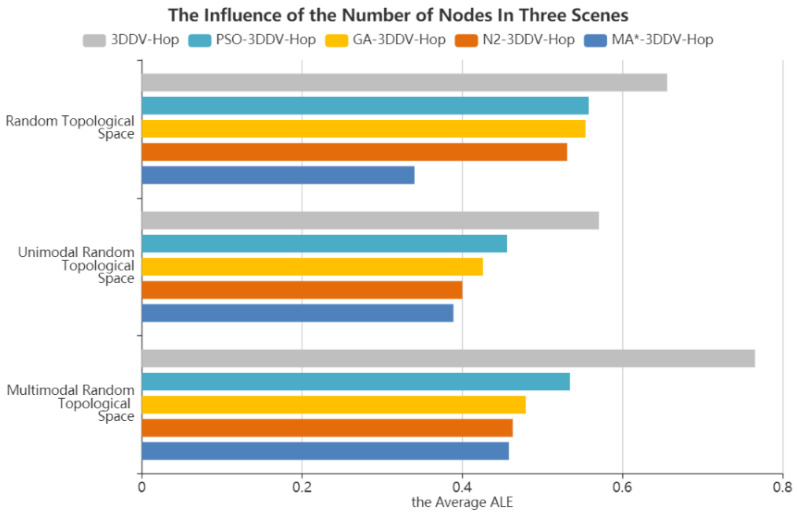
The average ALE of five algorithms in three scenes.

In the three scenes, the number of nodes is set between 70 and 130, the communication radius is 30 m, and the number of beacon nodes is 25. The experimental results are shown in Figure 13 and Figure 14.

It can be seen from Figure 13 that, in the three network topology scenes, the ALE of the five localization algorithms mentioned above decreases gradually with the increase of the number of nodes in the network. For the five algorithms, MA*-3DDV-Hop shows obvious advantages in three topological scenes, ALE value reaches the minimum, especially in random topological scene, where ALE value remains stable. In unimodal random topology scene, when the number of nodes increases to 80, MA*-3DDV-Hop algorithm achieves better ALE value compared with other algorithms; while in a multi-peak random topology scene, when the number of nodes reaches 110, the MA*-3DDV-Hop algorithm exceeds other algorithms to achieve a better ALE value.

Figure 14 shows the influence of the number of nodes on the average ALE of the five algorithms in the three scenes. It can be seen from the figure that compared with 3DDV-Hop, the average ALE of PSO-3DDV-Hop, GA-3DDV-Hop, N2-3DDV-Hop, and MA*-3DDV-Hop reduces by 14%, 15%, 19%, and 48% respectively in the random topology scene. Compared with 3DDV-Hop, the average ALE of PSO-3DDV-Hop, GA-3DDV-Hop, N2-3DDV-Hop, and MA*-3DDV-Hop reduces by 20%, 25%, 29%, and 31% in the unimodal network random topology scene. While in the multi-peak network random topology scene, the average ALE of PSO-3DDV-Hop, GA-3DDV-Hop, N2-3DDV-Hop, and MA*-3DDV-Hop reduces by 30%, 37%, 38%, and 39% respectively, compared with 3DDV-Hop.

As shown in Figure 13 and Figure 14, the localization error of wireless sensor network nodes can be narrowed by increasing the number of nodes in three different scenes. In the scenes of random topology and unimodal random topology, MA*-3DDV-Hop has striking advantages over other algorithms in terms of ALE and average ALE as the number of nodes increases. In multi-peak topology scene, ALE of MA*-3DDV-Hop is less affected by the increase of the number of nodes, while the average ALE is better than other algorithms. The results show that the MA*-3DDV-Hop algorithm has better stability and robustness when the number of nodes increases.

#### 5.4.3. The Influence of the Beacon Nodes

**Figure 15:**
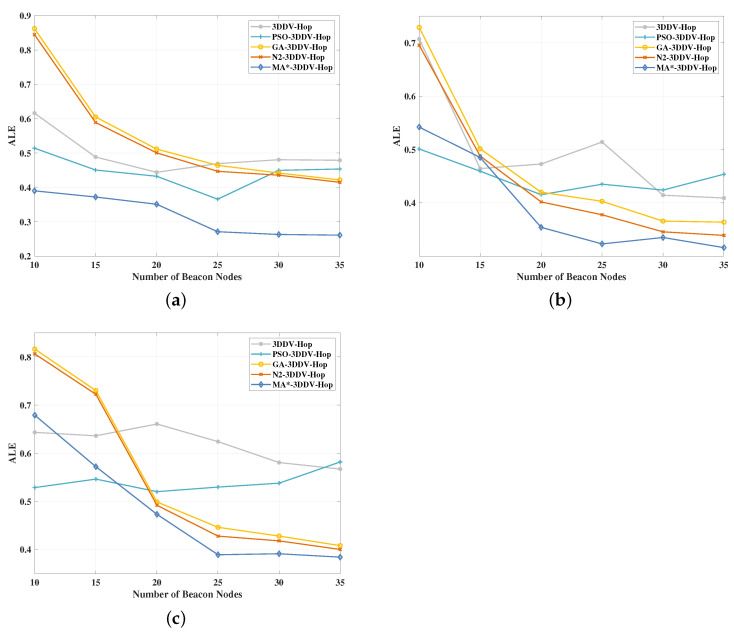
(**a**) The effect of the number of beacon nodes on ALE in a random topology scene. (**b**) The effect of the number of beacon nodes on ALE in unimodal random topology scene.(**c**) The effect of the number of beacon nodes on ALE in a multi-peak random topology scene.

**Figure 16:**
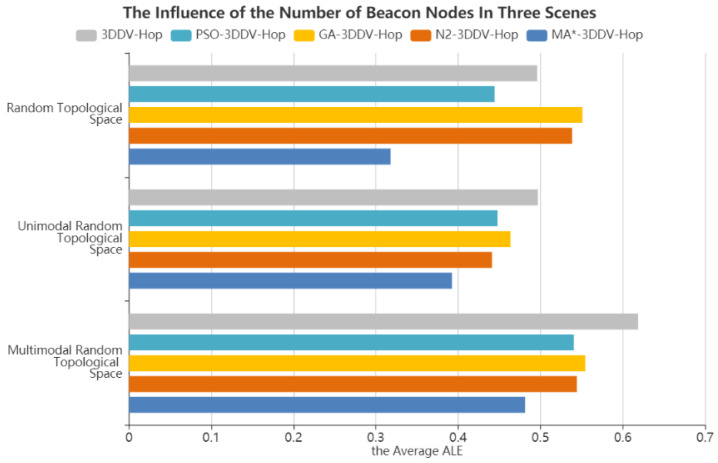
The average ALE of five algorithms in three scenes.

In the experiment, the number of beacon nodes is set to be between 10 and 35, the communication radius is 30 m, and the number of nodes is 130. The experimental results are shown in Figure 15 and Figure 16.

It can be seen from Figure 15 that, in the three network topology scenes, the ALE of the five localization algorithms mentioned above decreases gradually with the increase of the number of beacon nodes. Among these five algorithms, the MA*-3DDV-Hop algorithm also achieves obvious effects in three random topological scenes. In random topology scene, the ALE value of MA*-3DDV-Hop algorithm remains relatively minimal and stable with the increase of the number of nodes. In the scenes of unimodal and multi-peak random topology, the ALE value of MA* -3DDV-HOP algorithm is relatively high due to the insufficient proportion of early-stage beacon nodes. However, when the number of beacon nodes is greater than 17, the ALE of MA* -3DDV-HOP algorithm is the smallest.

Figure 16 shows the influence of the number of beacon nodes on the average ALE of the five algorithms in the three scenes. It can be seen from the figure that, compared with 3DDV-Hop, the average ALE of PSO-3DDV-Hop reduces by 10%, GA-3DDV-Hop rises by 11%, N2-3DDV-Hop rises by 8%, and MA*-3DDV-Hop reduces by 35%. Compared with 3DDV-Hop, the average ALE of PSO-3DDV-Hop, GA-3DDV-Hop, N2-3DDV-Hop and MA*-3DDV-Hop reduces by 9%, 6%, 11% and 21% respectively in the unimodal random topological scene. While in the multi-peak network random topology scene, the average ALE of PSO-3DDV-Hop, GA-3DDV-Hop, N2-3DDV-Hop, and MA*-3DDV-Hop reduces by 12%, 10%, 12%, and 22% respectively, compared with 3DDV-Hop.

As shown in Figure 15 and Figure 16, the localization error of wireless sensor network nodes can be decreased by increasing the number of beacon nodes in three different scenes. The MA*-3DDV-Hop algorithm achieves good effects in three kinds of topological environments, which reflects its adaptability and stability to the environment. In the scenes of random topology and unimodal random topology, MA*-3DDV-Hop has striking advantages over other algorithms in terms of ALE and average ALE as the number of beacon nodes increases.

The results show that the MA*-3DDV-Hop algorithm has better robustness and localization performance when the number of beacon nodes increases.

## 6. Performance Analysis

**Figure 17:**
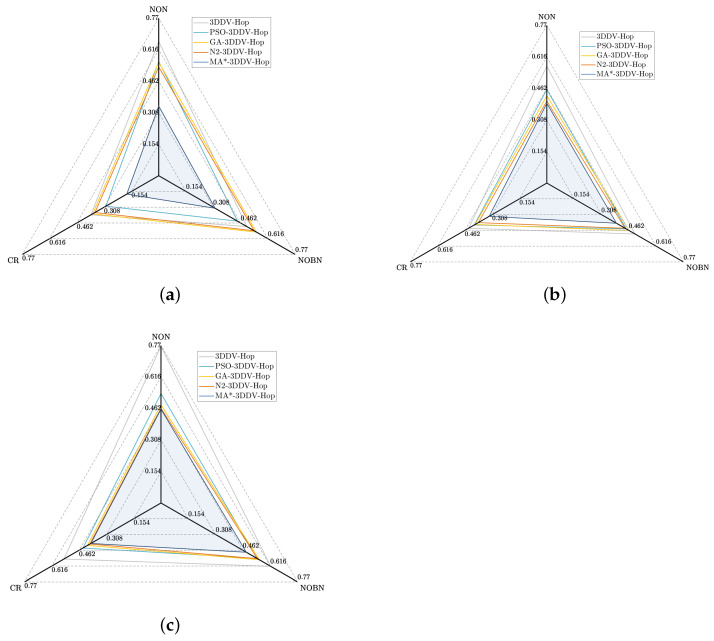
(**a**) Direct view of the influence of three factors of random topology scene. (**b**) Direct view of the influence of three factors on unimodal random topology scene. (**c**) Direct view of the influence of three factors on multi-peak random topology scene.

In order to verify the performance of our algorithm, the communication radius(CR), the number of beacon nodes(NOBN) and the number of nodes(NON) are comprehensively analyzed in three different scenes, as shown in Figure 17.

It can be intuitively seen the comprehensive performance of each algorithm in three different scenes, as well as which factors have a greater impact on each algorithm. The shaded parts In figures (a), (b), and (c) all represent the comprehensive performance of MA*-3DDV-Hop. In Figure 17a, based on the shape of each algorithm graph, we know that the three corners of the graph corresponding to MA*-3DDV-Hop algorithm are closest to the center point respectively, indicating that the MA*-3DDV-Hop algorithm is less affected by these three factors, and its comprehensive performance is superior. In Figure 17b, MA*-3DDV-Hop algorithm is affected by the number of nodes in the same degree as other algorithms, while the influence of communication radius and beacon node number is relatively small, and the comprehensive performance of the algorithm is also outstanding. In Figure 17c, the performance of MA*-3DDV-Hop algorithm is similar to that of other algorithms in terms of communication radius and the number of nodes, however, the affect of the number of beacon nodes is relatively small and the algorithm performance is better.

## 7. Conclusions and Future Work

In this paper, MA*-3DDV-Hop algorithm is proposed based on the shortcomings of large deviation and low precision in 3D complex scene. The algorithm combines the improved A* algorithm with DV-Hop algorithm to redefine the hop-count value path of the node, and uses the normalized value to correct the error caused by the average distance per hop. The multi-objective NSGA-II algorithm is used to optimize the estimated coordinates and obtain the optimal solution. On this basis, the errors produced in the above steps are corrected respectively, so as to minimise the cumulative error after superposition and achieve good results. In order to test the actual advantages, accuracy, and reliability of MA*-3DDV-Hop algorithm in three different scenes, this paper designs a comparative experiment with different algorithm models. The results show that the proposed method has good robustness, can achieve low localization error, and is better than the original DV-Hop and the related algorithms mentioned.

Future research directions can focus on the following issues: Most of the localization algorithms in sensor networks are based on the fact that the nodes are in static state, which can not be used in some application scenes that need mobile detection. How to build a comprehensive algorithm, intelligent localization according to the needs of the scene, ensure the localization accuracy, and build a complete algorithm system is worth considering.

## Figures and Tables

**Table 1:** Parameter table.

Parameter	Value
Angle weight of random space, $\varpi_{1}$	0.81
Hop weight of random space, $\varpi_{2}$	0.19
Angle weight of unimodal topology scene, $\varpi_{1}$	0.76
Hop weight of unimodal topology scene, $\varpi_{2}$	0.24
Angle weight of multi-peak topology scene, $\varpi_{1}$	0.68
Hop weight of multi-peak topology scene, $\varpi_{2}$	0.32
Correction factor, λ	0.1 (0.05–1.5)
Angle factor, θ	90 (70–90)
Constraint radius, r	10 (5–15)
The number of nodes, M	130 (70–130)
The number of beacon nodes, N	25 (10–40)
Communication radius, Rc	30 (25–50)
The number of iterations, NI	100
Stop iterations, SI	100
Pareto fraction, PF	0.3
Population size, P	100
Fitness value deviation, F	1e-100

## Data Availability

The data are not publicly available due to this research is still in progress.
